# Scoring Amino Acid Mutations to Predict Avian-to-Human Transmission of Avian Influenza Viruses

**DOI:** 10.3390/molecules23071584

**Published:** 2018-06-29

**Authors:** Xiaoli Qiang, Zheng Kou, Gang Fang, Yanfeng Wang

**Affiliations:** 1 Institute of Computing Science and Technology, Guangzhou University, Guangzhou 510006, China; qiangxl@mail.scuec.edu.cn (X.Q.); yuxiangqd@163.com (G.F.); 2 Henan Key Lab of Information-Based Electrical Appliances, College of Electrical and Electronic Engineering, Zhengzhou University of Light Industry, Zhengzhou 450002, China; wangyanfeng@zzuli.edu.cn

**Keywords:** avian influenza virus, interspecies transmission, amino acid mutation, machine learning

## Abstract

Avian influenza virus (AIV) can directly cross species barriers and infect humans with high fatality. Using machine learning methods, the present paper scores the amino acid mutations and predicts interspecies transmission. Initially, 183 signature positions in 11 viral proteins were screened by the scores of five amino acid factors and their random forest rankings. The most important amino acid factor (Factor 3) and the minimal range of signature positions (50 amino acid residues) were explored by a supporting vector machine (the highest-performing classifier among four tested classifiers). Based on these results, the avian-to-human transmission of AIVs was analyzed and a prediction model was constructed for virology applications. The distributions of human-origin AIVs suggested that three molecular patterns of interspecies transmission emerge in nature. The novel findings of this paper provide important clues for future epidemic surveillance.

## 1. Introduction

Wild birds are regarded as the natural reservoir of avian influenza virus (AIV) [1]. Interspecies transmission might have been enabled long ago, when wild birds were domesticated by humans. A highly pathogenic subtype of AIV, avian influenza H5N1, originated in Asia in 1996 [2]. Human-origin H5N1 virus was first isolated from clinical samples in 1997, confirming that the H5N1 virus can directly cross species barriers and fatally infect the respiratory system [3,4]. Human infection by H5N1 has been continuously reported since 2003, attracting the attention of both researchers and wider society [5,6,7,8]. Moreover, viral subtypes other than H5N1 can infect humans by direct interspecies transmission. Two infectious cases of H9N2 virus have been reported; one in 1999, the other in 2003 [9,10]. H7N7 virus infected farmers in the Netherlands in 2003 [11], and H7N9 has continuously infected China’s population since 2013 [12,13].

Interspecies transmission of AIV from its natural reservoir occurs in three steps: (1) the residence of AIVs in their wild animal hosts; (2) AIV contact with humans and direct infection with low probability; and (3) adaptation of AIVs to their new host and efficient human-to-human transmission thereafter. Thus far, AIV has not progressed beyond step 2, which represents initial adaption to the new host and low efficiency of transmission among the new host. The subtype viruses that can cross the species barrier and cause epidemics should be identified. Approximately twenty years has passed since human-originated AIV was first isolated from human samples in 1997. During this period, vast amounts of genomic data have accumulated in public databases. Therefore, after screening the important amino acid sites in the 11 viral proteins, the AIV risk can be predicted by machine learning methods and other mathematic models in the field of bioinformatics [14,15,16,17,18].

AIV transmission relies on amino acid mutations [19,20,21]. In a previous study, five amino acid factors (AA factors) summarized from 491 highly redundant amino acid attributes were associated with specific physiochemical amino acid properties, namely, polarity, secondary structure, molecular volume, codon diversity, and electrostatic charge [22]. In this paper, we use five AA factors to transform viral proteins and use the random forest (RF) method to select features from high-dimensional protein data and score them by their contributions to the data category. After ranking and screening the positions containing important mutation information, the classifier can predict the interspecies transmission phenotypes.

Two prediction models of AIVs have been published in the literature [23,24,25]. However, both of these models lack the protein data of hemagglutinin (HA) and neuraminidase (NA), and the biological meanings of the features were not clarified. To construct a more robust and meaningful model, we revise these models and screen the signature amino acid positions in HA, NA, and nine other viral proteins. To this end, we first identify 183 signature mutation positions by RF scoring, then predict AIV occurrence by four popular machine learning methods. Using the most effective classifier, we seek the important amino acid factors and the minimal range of signature positions. The study results will benefit epidemic surveillance and future studies on interspecies AIV transmission.

## 2. Results

### 2.1. Dataset

The cleaned dataset contained 869 high-quality AIV strains: 440 avian-origin AIVs (negative samples; H1–H14, H16 subtypes) and 429 human-origin AIVs (positive samples; H5N1, H5N6, H7N3, H7N7, H7N9, and H9N2 subtypes). The information related to these strains is summarized in Table S1.

### 2.2. Signature Amino Acid Residues

**Figure 1:**
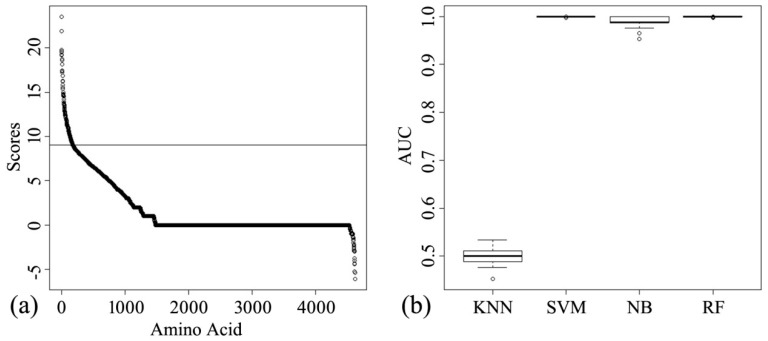
Importance score curve and the performances of *k*-nearest neighbor (KNN), support vector machine (SVM), naïve Bayes (NB), and random forest (RF) classifiers. (**a**) The ranked scores were calculated from five AA factors using the random forest method. The *x* and *y* coordinates denote the total length of the 11 protein alignments and the importance scores, respectively. The cutoff value (9) is indicated by the thin horizontal line. (**b**) Performances of the four classifiers were evaluated from 100 repeats of 10-fold cross-validation. The area under the curve (AUC) ranges from 0 to 1.

The importance score at each position in the 11 viral proteins was computed by RF. As shown in Figure 1a, the slope of the curve suddenly changes at an importance score of 9. Therefore, 9 was selected as the cutoff score, providing 183 signature positions for further machine learning.

As shown in Table 1, the HA protein contained the largest number of signature positions (65 amino acid residues), suggesting that HA is very important for interspecies transmission of AIVs. HA is mainly involved in receptor-binding and fusion activities. Positions HA102–HA290 (Table 1) locate in or close to the region of host receptor binding [26,27], and H163 is reportedly related to the specificity of receptor binding [28]. HA91, HA96, HA328, HA377, and HA397 locate at or near the fusion peptide [29], which triggers fusion activity in acidic environments and favors transmission to humans. The four HA327 positions located in the cleavage site are important virulence sites [30].

NA protein contains 44 signature positions (Table 1). The three NA52s located in the stalk deletion region are related to the virulence and pathogenesis of H5N1 influenza A virus [31]. NA19–NA37 located in the N-terminal are associated with structural stability and enzyme activity [32]. The PB2 627 position has been implicated in increased replication or virulence of AIVs in mammals [33]. PB1 14, located in the binding region of polymerase, is related to viral genome replication [34]. M2 97, which is affiliated with viral particle ensembles [35], was also screened. NEP 14, NP 373, and NP 377 are reportedly involved in intracellular transport of viral proteins [36,37].

The AA factors and RF method screened 183 signature positions, some of which are reported to be associated with the mechanism of interspecies transmission. All of the residues were useful, not only for constructing the prediction model but also for further investigating the molecular mechanisms underlying the interspecies transmission of AIVs.

### 2.3. Performance of the Prediction Model

The performances of the four classifiers are presented as boxplots in Figure 1b. The results were obtained from 100 repeats of 10-fold cross-validation. The area under the curve (AUC) medians in the support vector machine (SVM) and RF classifiers were almost 1. The AUC was clearly lower in the *k*-nearest neighbor (KNN) classifier, possibly because of the nonlinear prediction rules. Although the naïve Bayes (NB) classifier achieved a similar AUC score to the SVM classifier, its performance was poorer and less stable than those of the SVM and RF classifiers. Considering the complexity of the computation, the SVM classifier was selected as the optimal machine learning model for predicting avian-to-human transmission of AIVs.

### 2.4. Contributions of the AA Factors 

**Figure 2:**
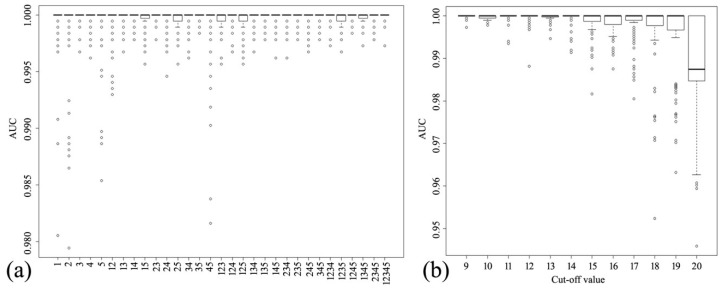
Contributions of AA factors and different mutation sets. (**a**) Performance of SVM classifier for different combinations of the five AA factors. The *x* and *y* coordinates denote the 31 combination patterns and the AUC values (from 0 to 1), respectively. Along the x axis, ‘13’ denotes that the set of 183 amino acid residues was transformed using AA Factor 1 and AA Factor 3 together, for example. (**b**) Contributions of mutation positions for different cutoff values (range 9–20). The *y* coordinate shows the AUC values.

The AIV strains were characterized by five AA factors. To understand the mechanism of interspecies transmission, the performance of the SVM classifier was calculated for all combination patterns of these AA factors. The result reveals the importance of the five AA factors. Most of the stable performances of the SVM classifier were contributed by AA Factor 3 or AA Factor 4 (Figure 2a). Notably, the median AUC values were almost 1 and remained stable under AA Factor 3 or AA Factor 4 alone. The SVM classifiers were unstable under AA Factor 1, AA Factor 2, and AA Factor 5 alone. Moreover, AA Factor 3 yielded a slightly better result than AA Factor 4. These results indicate an important role for AA Factor 3 in the avian-to-human transmission of AIVs. Therefore, AA Factor 3 was employed in further analysis.

### 2.5. Contributions of Mutation Positions at Different Cutoff Values

As mentioned above, 183 mutation sites survived a cutoff value of 9. To further explore the mechanism of interspecies transmission, we should reduce the range of crucial positions. To this end, the cutoff value was incremented in steps of 1 (thereby decreasing the number of mutation sites), and the performance of the SVM classifier was calculated with the five AA factors. As shown in Figure 2b, the SVM classifier achieved stable and high performance at cutoffs up to 14. The SVM classifier destabilized at higher cutoffs.

**Figure 3:**
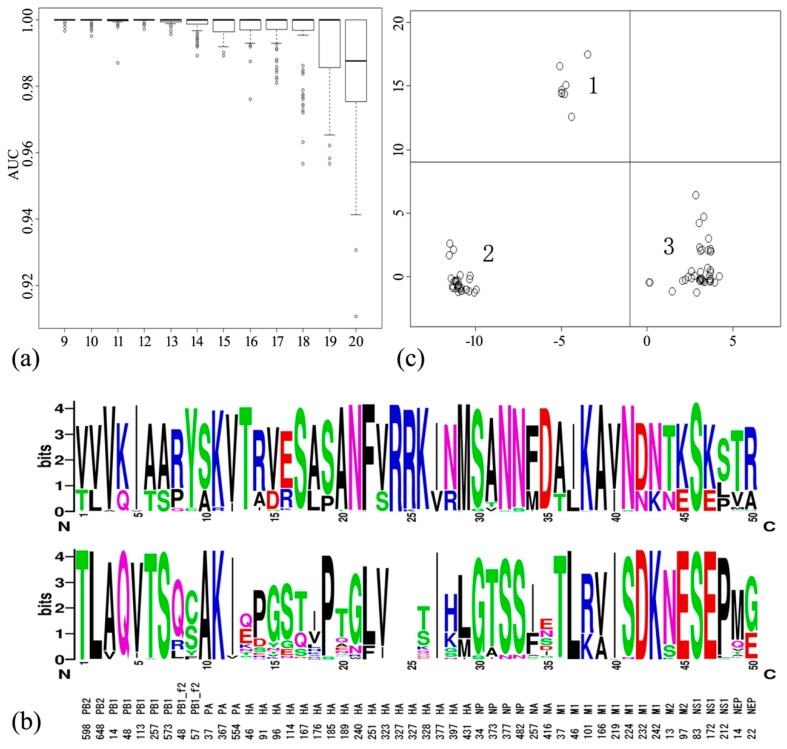
Minimal amino acid set for predicting AIVs. (**a**) Contributions of reduced mutation position sets. The *x* and *y* coordinates denote the cutoff (range 9–20) and the AUC values (range 0–1), respectively. (**b**) Profiles of 50 signature positions from human-origin (top) and avian-origin (bottom) AIVs. (**c**) Three patterns of human-origin AIVs clustered by the multidimensional scaling (MDS) method.

Considering the results under AA factor combinations and cutoff values, the performance of the SVM classifier with AA Factor 3 alone was assessed for different cutoffs. In this situation, the SVM classifier performed stably and well up to a cutoff of 13 (Figure 3a). The analysis results confirm that 13 is the extreme cutoff, giving 50 signature positions (Figure 3b). This set was regarded as the minimal mutation position set for predicting AIVs. We transformed these 50 signature residues using AA Factor 3 alone, and obtained the patterns of the human-origin AIVs (positive samples) by the multidimensional scaling method (Table S2). The resulting clusters are shown in Figure 3c. Cluster 1 comprises three H9N2 viruses (A/Hong Kong/1073/1999; A/Korea/KBNP-0028/2000; A/Bangladesh/0994/2011), two H7N3 viruses (A/Canada/rv504/2004; A/Mexico/InDRE7218/2012), two H7N7 viruses (A/Netherlands/219/2003; A/Italy/3/2013), and one H5N1 virus (A/Hong Kong/482/1997). Cluster 2 includes only H5N1 viruses isolated from 2003 to 2015. Cluster 3 is composed of H7N9 viruses, two H5N6 viruses (A/Yunnan/14563/2015; A/Yunnan/0127/2015), and two H9N2 viruses (A/Hong Kong/308/2014; A/Hunan/44558/2015). The distribution of the human-origin AIVs suggests that three molecular patterns of avian-to-human interspecies transmission emerge in nature.

## 3. Discussion

Avian influenza viruses can cross the species barrier, potentially causing a human pandemic. In this paper, human AIV transmission was predicted by a machine learning model with excellent performance (namely, SVM). We firstly screened 183 mutation positions in 11 viral proteins after ranking them by random forest (RF). Most of the screened amino acid positions locate in the important functional regions of receptor binding, fusion peptides, intracellular transport, protein active sites, or virus assembly [26,27,28,29,30,31,32,33,34,35,36,37]. Some of the residues at these positions have been related to interspecies transmission in earlier reports, such as HA102–HA290 [26,27], H163 [28], HA91, HA96, HA328, HA377 and HA397 [29], HA327 [30], NA52 [31], and PB2 627 [33]. The signature positions guarantee the accuracy of the classifier and are biologically meaningful, which will benefit epidemic surveillance and further studies on interspecies AIV transmission. The proposed method provides important clues for future surveillance and is a useful pre-screening tool for phenotype screening in high-level biological safety laboratories.

The signature positions related with the phenotype of interspecies transmission were screened by the method of random forest. Some yielded a modest score (PB2 627, for example). PB2 E627K was firstly identified in a mouse model [33] and found in the protein of other human-origin avian influenza viruses [12]. In part of the PB2 protein of the human seasonal influenza virus from the public database, PB2 E627 still existed. It is possible that the mutation PB2 E627K is not a strong marker for interspecies transmission, which is consistent with our results. In the future, we need to update the model with new molecular evidence in the field of virology and with more powerful technology in the field of machine learning.

Amino acid mutations in the HA protein are essential for AIV transmission in mammals [21], but mutations in other viral proteins are also necessary [19,20]. Mutations of different proteins introduce synergy and nonlinearity in interspecies transmission. This concept was supported by the present study. Specifically, the linear classifier (the KNN model) showed poor predictive performance on the initial set of 183 signature positions. Moreover, the minimal signature position set was 50 amino acid long and distributed among different viral proteins. This synergistic effect should be notable in further study.

The molecular characteristics of AA Factor 3 are related to molecular size or volume with high factor coefficients of bulkiness, residue volume, average volume of buried residues, side chain volume, and molecular weight [22]. Molecular size or volume is strong related with the binding of biology molecules, such as viral surface protein, host receptor, enzyme, and substrate. In this paper, the AA Factor 3 makes an important contribution to the prediction in terms of high accuracy, which agrees with previous results concerning the receptor binding of viral surface protein [26,27,28], enzyme activity of viral neuraminidase [32], and RNA binding of viral polymerase [34]. The slightly poor performance of other factors may suggest that host receptor binding, virus partial release triggered by viral neuraminidase, and viral polymerase activity play key roles for the interspecies transmission of avian influenza virus.

The patterns of human-origin AIVs were clarified by the MDS method. Cluster 1 was composed of one H5N1 virus from 1997; three H9N2 viruses from 1999, 2000, and 2011; two H7N3 viruses from 2004 and 2012; and two H7N7 viruses from 2003 and 2013. Cluster 2 contained only H5N1 viruses isolated from 2003 to 2015. Cluster 3 contained H7N9 viruses, two H5N6 viruses from 2015, and two H9N2 viruses from 2014 and 2015. The distribution of human-origin AIVs implies that three molecular patterns of avian-to-human interspecies transmission have emerged. Further investigations on the appearance of novel patterns should be undertaken in future.

The proposed method is applicable to other infectious pathogens that can cross species barriers. As deep learning technology develops, powerful methods that omit feature selection and complex computations might emerge. To better understand the interspecies transmission mechanism of AIVs, the prediction model could be supplemented with information on the host’s genetic background [38].

## 4. Materials and Methods 

### 4.1. Dataset 

**Figure 4:**
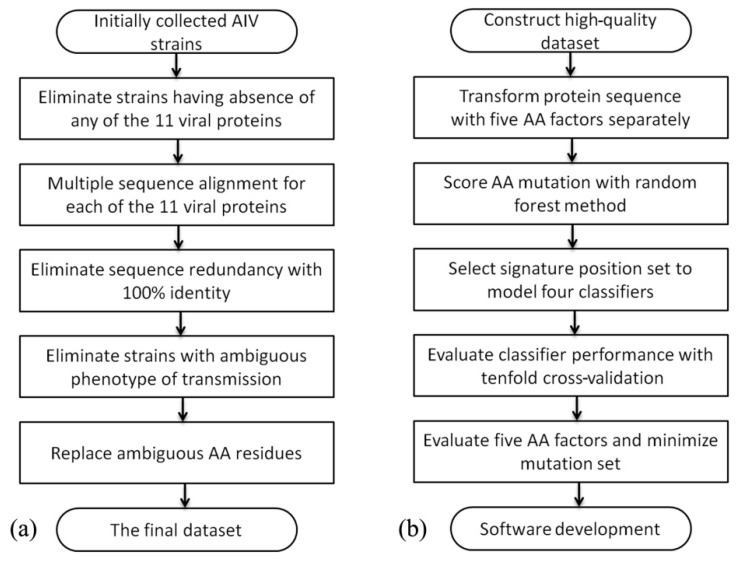
Flowchart of methods used in this paper. (**a**) High-quality dataset construction; (**b**) Machine learning algorism.

The avian- and human-origin AIVs were collected from the EpiFlu public database (http://platform.gisaid.org/epi3/frontend) and processed using multiple public bioinformatics tools and algorithms (Figure 4). The details of each procedure are described below.

Step 1: In total, 6305 avian-origin and 644 human-origin AIV strains were obtained from the public influenza virus database. The strains were isolated between January 1996 and February 2016. GISAID deposits high-quality genomic sequences along with their clinical information.

Step 2: Our prediction classifiers were based on eleven viral proteins (PB2, PB1, PBI-F2, PA, HA, NP, NA, M1, M2, NS1, and NEP) with reported roles in interspecies transmission. AIV strains lacking any of these 11 protein sequences in the GISAID database, and strains without subtype information, were excluded in this step.

Step 3: The amino acid residues in the 11 proteins were numbered by the multiple sequence alignment tool MUSCLE [39], using the seasonal human H3 subtype virus as the reference. This step eliminated strains lacking more than 3 amino acids at any protein terminal. The missing residues were replaced by the corresponding residues in the protein sequence with highest identity.

Step 4: To reduce redundancy in the dataset, the AIV strains should differ by at least one amino acid. The amino acid sequences were compared using the CD-Hit tool [40].

Step 5: If the genome sequences of the avian-origin and human-origin AIV strains share high identity, the interspecies transmission capabilities of the avian-origin strains are ambiguous. Therefore, this step eliminated avian-origin strains in which any nucleotide sequence of the eight genome segments shared > 97% identity with that of the human-origin strains. The elimination was performed by the BLAST + tool [41].

Step 6: Ambiguous amino acid residues such as ‘X’ and ‘B’ were replaced by the corresponding residues in the protein sequence with highest identity.

The final dataset for predicting AIV interspecies transmission contained 429 positive samples (human-origin AIVs) and 440 negative samples (avian-origin AIVs). All of these strains are listed in Table S1.

### 4.2. Recognition of Signature Positions 

The random forest method is very popularly used for feature selection of prediction problems and can rank the importance of the features in a large scale to discriminate the different categories. The signature positions in the 11 viral proteins were recognized by the RF method (RF, https://cran.r-project.org/web/packages/randomForest/index.html). In each strain, the 11 proteins were concentrated in the following order: PB2 > PB1 > PB1-F2 > PA > HA > NP > NA > M1 > M2 > NS1 > NEP. The proteins with the length of 4620 amino acids were then transformed into numerical sequences of the amino acid factor. Any deletions or insertions in the protein were replaced by zeros. The strains were processed sequentially and accumulated into the total dataset, which was input to the RF. The positive samples (human-origin AIVs) and negative samples (avian-origin AIVs) were classified by their importance scores at each amino acid position. As the classification was based on five factors, the final importance score at each position was the sum of five calculations. Therefore, highly scoring positions were important for distinguishing positive and negative samples. These high scorers were regarded as important amino acid mutations in the interspecies transmission of AIVs. Breiman’s random forest algorithm was used as default.

### 4.3. Constructing the Classifier Model 

The machine learning method can solve the classification problem and the numeric features of the positive and negative samples are essential for classification. After screening the signature positions as mentioned above, each strain was represented by an amino acid residue set. These amino acid sets were again transformed into numerical sequences of the five AA factors. Each strain was represented as a numeric vector of length 5N, where N is the number of amino acids in an amino residue set. The interspecies AIV transmission was then predicted by four popular machine learning models that are widely used in bioinformatics and computational biology: (1) support vector machine (SVM, https://cran.r-project.org/web/packages/e1071/index.html), (2) random forest (RF, https://cran.r-project.org/web/packages/randomForest/index.html), (3) naïve Bayes (NB, https://cran.r-project.org/web/packages/e1071/index.html), and (4) *k*-nearest neighbor (KNN, https://cran.r-project.org/web/packages/class/index.html). The present prediction task is a two-class classification problem (in which human-origin and avian-origin AIVs are classified as positive and negative, respectively). The four classifiers were implemented in the R environment and related packages.

The SVM classifier performs the classification in a high-dimensional feature space, which was transferred from the input feature vector with the kernel function. If the samples from two categories were partly overlapped in the original feature space, the SVM will have good performance. In this paper, the optimal hyperplane is determined with the regularization parameter C (C = 1) and the radial basis function (RBF) as default. The RF classifier is an ensemble of many decision trees. Each tree is fully grown using part of the samples in the training dataset selected with the bootstrap technique. The NB is constructed based on the Bayes theorem. Both RF and NB were implemented with the default parameter in the package. The KNN classifier is a nonparametric method to determine a sample category by a majority vote of its neighbors; the number of neighbors in this paper was set to be 3 (*k* = 3).

### 4.4. Evaluating the Performance of Different Classifiers

The four classifiers were trained on 387 positive samples and 396 negative samples randomly selected from the AIV dataset. The remaining 10% of samples (42 positive and 44 negative samples) were reserved as an independent test dataset for assessing the performances of the classifiers. The classifier performances were evaluated by 10-fold cross-validation and the receiver operating characteristic (ROC) curve. The area under the ROC curve (AUC) reveals the optimal parameters in the four classifiers. To compare the classifier performances, we repeated the evaluation process 100 times and plotted the distributions of the resulting AUC values. The ROC curve relates the true and false positive rates, where both rates range from 0 to 1. The AUC was calculated by the ‘ROCR’ package in R (https://cran.r-project.org/web/packages/ROCR/index.html). As both rates range from 0 to 1, AUC also ranges from 0 to 1. A higher AUC value denotes a higher performance of the classifier. The human-origin AIVs were shown by the multidimensional scaling method in R (MDS, https://cran.r-project.org/web/packages/MASS/index.html) and the amino acid profile was drawn by the WebLogo server (http://weblogo.berkeley.edu/logo.cgi).

### 4.5. Prediction Software

By integrating the features at the signature positions with the best-performing classifier, we constructed a software program for predicting avian-to-human transmission of AIVs (delivery by request).

## Figures and Tables

**Table 1:** Scores for the 183 signature amino acids of avian influenza viruses (AIVs).

Num	Pro ^1^	Pos ^2^	Score	Num	Pro	Pos	Score	Num	Pro	Pos	Score
1	PB2	389	11.95	62	HA	176	13.61	123	NA	65	10.98
2	PB2	478	9.81	63	HA	179	10.08	124	NA	66	9.93
3	PB2	598	17.36	64	HA	185	14.73	125	NA	72	10.96
4	PB2	627	9.83	65	HA	189	14.55	126	NA	79	11.38
5	PB2	648	15.55	66	HA	207	9.49	127	NA	85	9.57
6	PB2	676	9.94	67	HA	211	11.15	128	NA	88	10.13
7	PB1	14	19.16	68	HA	213	11.40	129	NA	100	11.34
8	PB1	48	18.13	69	HA	216	12.17	130	NA	187	10.48
9	PB1	113	18.58	70	HA	221	10.57	131	NA	205	9.62
10	PB1	149	11.09	71	HA	222	9.02	132	NA	233	10.13
11	PB1	257	13.74	72	HA	240	17.36	133	NA	249	9.05
12	PB1	383	12.14	73	HA	251	16.26	134	NA	257	17.24
13	PB1	384	9.34	74	HA	266	10.96	135	NA	265	9.29
14	PB1	387	11.50	75	HA	273	12.53	136	NA	285	10.46
15	PB1	525	9.95	76	HA	274	9.23	137	NA	287	10.65
16	PB1	573	13.38	77	HA	275	9.38	138	NA	288	10.28
17	PB1	628	9.59	78	HA	289	10.36	139	NA	333	10.07
18	PB1-F2	4	9.38	79	HA	290	11.74	140	NA	338	9.02
19	PB1-F2	26	9.24	80	HA	297	10.48	141	NA	347	9.82
20	PB1-F2	48	13.50	81	HA	315	11.98	142	NA	359	10.08
21	PB1-F2	50	11.81	82	HA	323	13.04	143	NA	368	11.05
22	PB1-F2	57	16.85	83	HA	327	12.84	144	NA	369	10.82
23	PB1-F2	77	11.29	84	HA	327	16.23	145	NA	399	11.71
24	PA	37	18.74	85	HA	327	19.25	146	NA	415	9.43
25	PA	61	12.34	86	HA	327	10.41	147	NA	416	13.74
26	PA	63	9.70	87	HA	328	16.24	148	NA	418	9.09
27	PA	129	9.34	88	HA	377	13.91	149	NA	445	12.13
28	PA	337	11.25	89	HA	397	16.18	150	NA	468	9.66
29	PA	356	12.77	90	HA	407	9.49	151	M1	15	9.79
30	PA	367	14.56	91	HA	431	13.52	152	M1	27	12.16
31	PA	405	10.01	92	HA	492	9.49	153	M1	37	14.66
32	PA	554	14.67	93	HA	495	11.15	154	M1	46	14.96
33	PA	607	11.97	94	HA	496	10.62	155	M1	101	13.28
34	PA	684	12.20	95	HA	500	11.88	156	M1	140	12.40
35	PA	712	9.25	96	HA	503	12.76	157	M1	142	11.31
36	HA	40	9.42	97	HA	526	11.91	158	M1	166	17.35
37	HA	42	9.21	98	HA	530	11.26	159	M1	205	11.09
38	HA	45	11.92	99	HA	531	11.67	160	M1	219	13.18
39	HA	46	16.27	100	HA	534	12.77	161	M1	224	23.52
40	HA	53	9.87	101	NP	34	17.45	162	M1	232	14.80
41	HA	57	9.42	102	NP	77	12.39	163	M1	242	19.59
42	HA	65	10.99	103	NP	105	10.61	164	M1	248	11.25
43	HA	66	11.13	104	NP	373	14.73	165	M2	13	13.66
44	HA	79	12.71	105	NP	377	21.88	166	M2	21	10.53
45	HA	81	12.03	106	NP	482	19.71	167	M2	97	15.79
46	HA	84	10.27	107	NA	19	9.20	168	NS1	77	10.59
47	HA	91	17.33	108	NA	23	11.02	169	NS1	80	12.48
48	HA	96	14.98	109	NA	37	9.57	170	NS1	81	12.55
49	HA	102	9.04	110	NA	41	11.30	171	NS1	82	12.01
50	HA	112	12.67	111	NA	42	9.33	172	NS1	83	14.52
51	HA	114	19.46	112	NA	47	10.12	173	NS1	84	10.21
52	HA	115	9.66	113	NA	48	11.23	174	NS1	172	14.21
53	HA	121	10.42	114	NA	49	10.85	175	NS1	179	11.18
54	HA	124	10.28	115	NA	50	9.14	176	NS1	197	9.32
55	HA	131	12.31	116	NA	52	12.38	177	NS1	212	14.19
56	HA	142	12.01	117	NA	52	10.34	178	NEP	14	13.01
57	HA	163	10.07	118	NA	52	9.75	179	NEP	22	15.38
58	HA	164	9.03	119	NA	53	9.03	180	NEP	40	10.28
59	HA	167	14.22	120	NA	58	11.05	181	NEP	60	9.17
60	HA	173	12.81	121	NA	60	9.34	182	NEP	100	10.58
61	HA	174	10.16	122	NA	63	9.44	183	NEP	115	11.10

^1^ Viral protein; ^2^ Position of amino acid residue as H3 subtype numbering.

## Supplementary Materials

Table S1: AIV Strains in the final dataset, Table S2: Human-origin AIVs clustered by the MDS method.
